# Cytokine and Complement Response in the Glaucomatous βB1-CTGF Mouse Model

**DOI:** 10.3389/fncel.2021.718087

**Published:** 2021-11-18

**Authors:** Sabrina Reinehr, Johanna D. Doerner, Ana M. Mueller-Buehl, Dennis Koch, Rudolf Fuchshofer, H. Burkhard Dick, Stephanie C. Joachim

**Affiliations:** ^1^Experimental Eye Research Institute, University Eye Hospital, Ruhr-University Bochum, Bochum, Germany; ^2^Institute of Human Anatomy and Embryology, University Regensburg, Regensburg, Germany

**Keywords:** glaucoma, βB1-CTGF, complement system, classical pathway, CXCL1, cytokines

## Abstract

Glaucoma is a complex neurodegenerative disease leading to a loss of retinal ganglion cells (RGCs) and optic nerve axons. An activation of the complement system seems to contribute to cell loss in this disease. Hence, we investigated a possible initiation of the complement system and the cytokine response in the βB1-CTGF glaucoma model. In these mice, intraocular pressure is elevated, which is the main glaucoma risk factor in patients, and RGC loss occurs at 15 weeks of age. Therefore, quantitative real-time PCR and immunohistological experiments were performed in 5-, 10-, and 15-week-old βB1-CTGF animals and their corresponding wildtypes (WT) to analyze the expression of several complement system factors. We could show that mRNA levels of the terminal complement pathway components *C3* and C5 (*Hc*) were upregulated at 10 weeks. In accordance, more C3^+^ and membrane attack complex^+^ cells were observed in transgenic retinae. Further, the C5a receptor anaphylatoxin receptor (*C5ar*) and the complement component C5a receptor 1 (*C5ar1*; CD88) mRNA levels were upregulated in 10- and 15-week-old βB1-CTGF mice. Interestingly, all three activation routes of the complement system were elevated in βB1-CTGF mice at some age. Especially C1q, as a marker of the classical pathway, was significantly increased at all investigated ages. Furthermore, mRNA expression levels of interferon-γ (*Infg*) were upregulated at 5 weeks, while *Cxcl1* and *Cxcl2* mRNA levels were upregulated at 10 and 15 weeks. The mRNA levels of the chemokines *Cxcl10* were increased at all ages in βB1-CTGF mice. These results lead to the assumption that in these transgenic mice, a complement activation mainly through the classical pathway as well as a cytokine response plays a major role in cell death.

## Introduction

The complement system forms an integral part of the early immune response. Three complement cascades, the classical, the lectin, and the alternative, can activate the terminal pathway including the membrane attack complex (MAC) (Walport, 2001; Bayly-Jones et al., 2017). It could be demonstrated that in nucleated target cells, membrane disruption leads to cell death by apoptosis or by lysis if enough MAC is present (Koski et al., 1983; Kim et al., 1989; Nauta et al., 2002).

In the eye, the complement system is usually activated at low levels and provides immune tolerance (Sohn et al., 2000; Niederkorn, 2007). Recent data indicates that all retinal cells are able to express components of the complement system (Pauly et al., 2019). Albeit it resembles a double-edged sword since its activation can either benefit or harm the host and is therefore probably involved in several retinal diseases. In the last years, a contribution of the complement in glaucoma, a leading cause of blindness worldwide, was described. An elevated intraocular pressure (IOP) is the main risk factor for this disease and hallmarks are a loss of retinal ganglion cells (RGCs) as well as a degeneration of optic nerve axons (Casson et al., 2012). In donor eyes of glaucoma patients, an upregulation of proteins associated with the complement system, for example C1s, C3, C9, and mannose-binding serin proteases (MASPs) 1 and 2, were detected (Tezel et al., 2010; Doudevski et al., 2014). Furthermore, C1q, a component linked to the classical pathway, was found in human glaucoma retinae (Stasi et al., 2006). In addition, proteome analyses identified lectin pathway proteins (Tezel et al., 2010). Assembling of MAC can result in an activation of pro-inflammatory cytokines, such as interleukin (IL)-1, tumor necrosis factor α (TNFα), CCL2, interferon-γ (INF-γ), or IL-8 (CXCL8) (Schonermark et al., 1991; Liu et al., 2011; Xie et al., 2019). In aqueous humor samples of glaucoma patients, certain cytokines, like IL-1α, IL-8, CCL2, and INF-γ, were upregulated (Chua et al., 2012; Chono et al., 2018). Furthermore, a recent study suggests that elevated levels of IL-8 can be considered a risk factor used for detecting and managing glaucoma (Chono et al., 2018).

In the DBA2/J model as well as in a surgical induced ocular hypertension (OHT) model, an activated complement system could be revealed (Kuehn et al., 2006; Stasi et al., 2006). Similar results were shown in an IOP-independent glaucoma model (Reinehr et al., 2016, 2018b). However, the contribution of the complement system in the transgenic βB1-CTGF mouse model has not been investigated yet. Previous studies showed that in these transgenic mice, IOP elevation is accompanied by a degeneration of optic nerve axons and apoptotic RGC loss making it a reliable model for primary open-angle glaucoma (POAG) (Junglas et al., 2012; Reinehr et al., 2019b; Weiss et al., 2021). Particularly, already at the ages of 5 and 10 weeks, more cleaved caspase 3^+^ RGCs and TUNEL^+^ apoptotic cells were detected in the transgenic βB1-CTGF mice. This was accompanied by a loss of retinal neurofilament H in 10-week-old mice (Weiss et al., 2021).

Hence, we now aimed to determine, which mechanisms lead to the apoptosis and subsequent cell loss in this model. Here, we investigated a possible contribution of the complement system. In addition, a potential corresponding cytokine response was investigated in βB1-CTGF mice at different ages. We revealed, for the first time, an activation of the complement system, mainly via the classical pathway, as well as a response of pro-inflammatory cytokines. This was noted even before a notably RGC loss occurred in the βB1-CTGF model as shown previously (Reinehr et al., 2019b).

## Materials and Methods

### Animals

All procedures concerning animals adhered to the ARVO statement for the use of animals in ophthalmic and vision research. All experiments involving animals were approved by the animal care committee of North Rhine-Westphalia, Germany. Mice were kept under environmentally controlled conditions with free access to chow and water.

The used βB1-CTGF and wildtype (WT) animals in this study had a CD1 background (Reinehr et al., 2019b; Weiss et al., 2021). All animals were bred in-house at the animal facility at the Ruhr-University Bochum. WT CD1 mice for breeding were obtained from Charles River (Sulzfeld, Germany). βB1-CTGF mice for breeding were kindly provided by Prof. Fuchshofer (University Regensburg, Germany). Then, all animals for this study were bred and housed at the animal facility at the Ruhr-University Bochum (Bochum, Germany). Potential βB1-CTGF mice were screened by isolating genomic DNA from tail biopsies and testing for transgenic sequenced by PCR, using the following primer sequences: 5′-GGAAGTGCCAGCTCATCAGT-3′ and 5′-GTGCGGGACAGAAACCTG-3′.

5-, 10-, and 15-week-old female and male mice were included in the current study.

### Quantitative Real-Time PCR

Both retinae of an animal at each age (5, 10, and 15 weeks; *n* = 5–7 animals/group) were pooled for RNA preparation and cDNA synthesis as previously described (Reinehr et al., 2019b). Total RNA was extracted with the Gene Elute Mammalian Total RNA Miniprep Kit, including a digestion with RNAase free DNase according to the manufacturer’s instructions (Sigma-Aldrich, St. Louis, MO, United States). The quantity and quality of the RNA were determined utilizing a NanoDrop ONE (Thermo Fisher Scientific, Schwerte, Germany). Total RNA of 1 μg was applied for reverse transcription using a cDNA synthesis kit (Thermo Fisher Scientific). The designed oligonucleotides for RT-qPCR are shown in Table 1. *Actb* and *Cyclophilin* (*Ppid*) served as reference genes. The RT-qPCR was performed using DyNAmo Flash SYBR Green (Thermo Fisher Scientific) on the PikoReal RT-qPCR Cycler (Thermo Fisher Scientific) (Palmhof et al., 2018; Wilmes et al., 2018). No template controls, where PCR grade water (Roche Diagnostics, Basel, Switzerland) was applied instead of cDNA, were used as negative controls (Adams, 2020). For quantification, values were transferred to REST© software (Qiagen, Hilden, Germany) for further analysis.

**TABLE 1:** Sequences of oligonucleotides.

**Gene**	**Forward (F) and reverse (R) oligonucleotides**	**GenBank acc. no.**	**Amplicon size**	**Primer efficiency**
*Actb*-F *Actb*-R	ctaaggccaaccgtgaaag accagaggcatacagggaca	NM_007393.5	104 bp	1.00
*C1qa*-F *C1qa*-R	cgggtctcaaaggagagaga tcctttaaaacctcggatacca	NM_007572.2	71 bp	1.00
*C1qb*-F *C1qb*-R	aggcactccagggataaagg ggtcccctttctctccaaac	NM_009777.3	80 bp	1.00
*C1qc*-F *C1qc*-R	atggtcgttggacccagtt gagtggtagggccagaagaa	NM_007574.2	75 bp	1.00
*C3*-F *C3*-R	accttacctcggcaagtttct ttgtagagctgctggtcagg	NM_009778.3	75 bp	1.00
*C5ar*-F *C5ar*-R	gctgatggtgggttttgtgt gttcagcttctccaccctct	AY220494.1	229 bp	0.94
*C5ar1*-F *C5ar1*-R	gctgatggtgggttttgtgt gttcagcttctccaccctct	NM_001173550.1	229 bp	0.85
*Cfb*-F *Cfb*-R	ctcgaacctgcagatccac tcaaagtcctgcggtcgt	M57890.1	112 bp	1.00
*Cfd*-F *Cfd*-R	atggagtgacggatgacgac gggzgaggcactacactctg	NM_013459.4	109 bp	1.00
*Cfh*-F *Cfh*-R	aaaaaccaaagtgccgagac ggaggtgatgtctccattgtc	NM_009888.3	74 bp	1.00
*Cxcl2*-F *Cxcl2*-R	tgaactgcgctgtcaatgc gcttcagggtcaaggcaaac	NM_009140.2	153 bp	1.00
*Cxcl1*-F *Cxcl1*-R	agactccagccacactccaa tgacagcgcagctcattg	NM_008176.3	130 p	1.00
*Cxcl10*-F *Cxcl10*-R	gctgccgtcattttctgc tctcactggcccgtcat	NM_021274.2	110 bp	1.00
*Hc*-F *Hc*-R	tgacaccaggcttcagaaagt agttgcgcacagtcagctt	XM_017315669.2	69 bp	1.00
*Infg*-F *Infg*-R	atctggaggaactggcaaaa ttcaagacttcaaagagtctgagg	NM_008337.4	89 bp	1.00
*Masp2*-F *Masp2*-R	ggcggctactattgctcct aacacctggcctgaacaaag	NM_001003893.2	86 bp	1.00
*Ppid*-F *Ppid*-R	ttcttcataaccacaagtcaagacc tccacctccgtaccacatc	M60456.1	95 bp	1.00

*The listed oligonucleotide pairs were used in quantitative real-time PCR experiments, while Actb and Cyclophilin (Ppid) served as housekeeping genes. The predicted amplicon sizes are given. F, forward; R, reverse; acc. no., accession number; bp, base pair.*

### Tissue Preparation for Immunohistology

At 5, 10, and 15 weeks, eyes (*n* = 7–9/group) were enucleated and fixed in 4% paraformaldehyde for 1 h. Thereafter, the eyes underwent a 30% sucrose treatment overnight and got embedded in a Neg-50 compound (Tissue-Tek; Thermo Fisher Scientific). 10 μm thick cross-sections were cut with a cryostat (Thermo Fisher Scientific) for further staining (Casola et al., 2016).

### Immunohistology

In order to identify different complement markers, specific immunofluorescence antibodies were applied (*n* = 7–9 eyes/group; 6 sections/animal, Table 2; Reinehr et al., 2016). Briefly, retina cross-sections were blocked with a solution containing 10–20% donkey, 2–3% BSA and/or goat serum and 0.1% or 0.3% Triton-X in PBS. Sections were incubated with primary antibodies at room temperature overnight. Incubation using corresponding secondary antibodies was performed for 1 h on the next day. Nuclear staining with 4′,6 diamidino-2-phenylindole (DAPI, Serva Electrophoresis, Heidelberg, Germany) was included to facilitate the orientation on the slides. Negative controls were performed for each stain by using secondary antibodies only.

**TABLE 2:** Primary and secondary antibodies used for immunohistology.

**Primary antibodies**					**Secondary antibodies**		
**Antibody**	**Company**	**Dilution**	**Manufacturer proof of validation**	**References**	**Antibody**	**Company**	**Dilution**
Anti-C1q	Abcam	1:200	Recombinant Anti-C1q antibody [4.8] KO Tested (ab182451) | Abcam	Welsh et al., 2020	Donkey anti-rabbit Alexa Fluor 555	Invitrogen	1:400
Anti-C3	Cedarlane	1:500	Anti-Rat C3, Purified, (pAb) (Rabbit Ig)—Cedarlane (cedarlanelabs.com)	Freeley et al., 2016	Goat anti-rabbit IgG Cy 3	Linaris	1:500
					Donkey anti-rabbit Alexa Fluor 488	Jackson Immuno Research	1:500
Anti-C5b-9 (MAC)	Biozol	1:100	C5b-9, Rat, mAb 2A1, Mouse IgG1 | BIOZOL	Reinhard et al., 2019	Goat anti-mouse Alexa Fluor 488	Life technology	1:500
Anti-Factor B	Quidel	1:1,000	Layout 1 (quidel.com)	Matsumoto et al., 1997	Donkey anti-goat Alexa Fluor 488	Dianova	1:500
Anti-GFAP	Millipore	1:2,000	Anti-Glial Fibrillary Acidic Protein Antibody| AB5541 (merckmillipore.com)	Ibrahim et al., 2011	Donkey anti-chicken Cy3	Millipore	1:500
Anti-Iba1	SySy	1:500	Synaptic Systems—IBA1 (sysy.com)	Su et al., 2019	Donkey anti-chicken Cy3	Millipore	1:500
Anti-IP10	Santa Cruz	1:100	Anti-IP-10 Antibody (E-2)| SCBT—Santa Cruz Biotechnology	Sen et al., 2020	Donkey anti-mouse Alexa Fluor 555	Abcam	1:500
Anti-MASP2	Biozol	1:400	MASP2 Polyclonal Antibody, IgG, Unconjugated, Rabbit| BIOZOL	Not applicable	Donkey anti-rabbit Alexa Fluor 555	Invitrogen	1:700
Anti-NeuN	Millipore	1:500	Anti-NeuN Antibody| ABN91 (merckmillipore.com)	Pichavaram et al., 2018	Donkey anti-chicken Cy3	Millipore	1:500
Anti-RBPMS	Millipore	1:200	Anti-RBPMS Antibody| ABN1362 (merckmillipore.com)	Rodriguez et al., 2014	Donkey anti-rabbit Alexa Fluor 488	Jackson Immuno Research	1:500

### Histological Examination

The single plane photographs were taken using a fluorescence microscope using 400x magnification (Axio Imager M1 or M2, Zeiss, Oberkochen, Germany). Two photos of the peripheral and two of the central part of each retina cross-section were captured. The images were transferred to Corel Paint Shop Pro (V13, Corel Corporation, Ottawa, Canada) and equal excerpts were cut out. Afterward, C3^+^, MAC^+^, Factor B^+^, and C1q^+^ cells were counted in the ganglion cell layer (GCL) using ImageJ software (NIH, Bethesda, MD, United States) (Reinehr et al., 2019b).

MASP2^+^ staining area in the nerve fiber layer, GCL, and inner plexiform layer, was evaluated using ImageJ software. Briefly, images were transformed into grayscale. To minimize interference with background labeling, a defined rolling ball radius of 150 pixels was subtracted. The percentage (%) of the labeled area was then measured between defined thresholds, which were obtained when the grayscale and the original picture corresponded the most (lower threshold: 13.76; upper threshold: 264.8) (Casola et al., 2016; Reinehr et al., 2018a).

### Statistics

Regarding RT-qPCR, the relative expression values are presented as median ± quartile + minimum/maximum and were assessed via Pair Wise Fixed Reallocation Randomization Test using REST© software (Qiagen) (Pfaffl, 2001; Reinehr et al., 2019b, c). Immunhistological data are presented as mean ± SEM. The βB1-CTGF animals were compared to the WT group via two-tailed Student’s *t*-test using Statistica Software (Version 13, Dell, Tulsa, OK, United States). Control values were set to 100%. *P*-values below 0.05 were considered statistically significant, with ^∗^*p* < 0.05, ^∗∗^*p* < 0.01, and ^∗∗∗^*p* < 0.001.

## Results

### Upregulation of *C3* mRNA Levels

**FIGURE 1:**
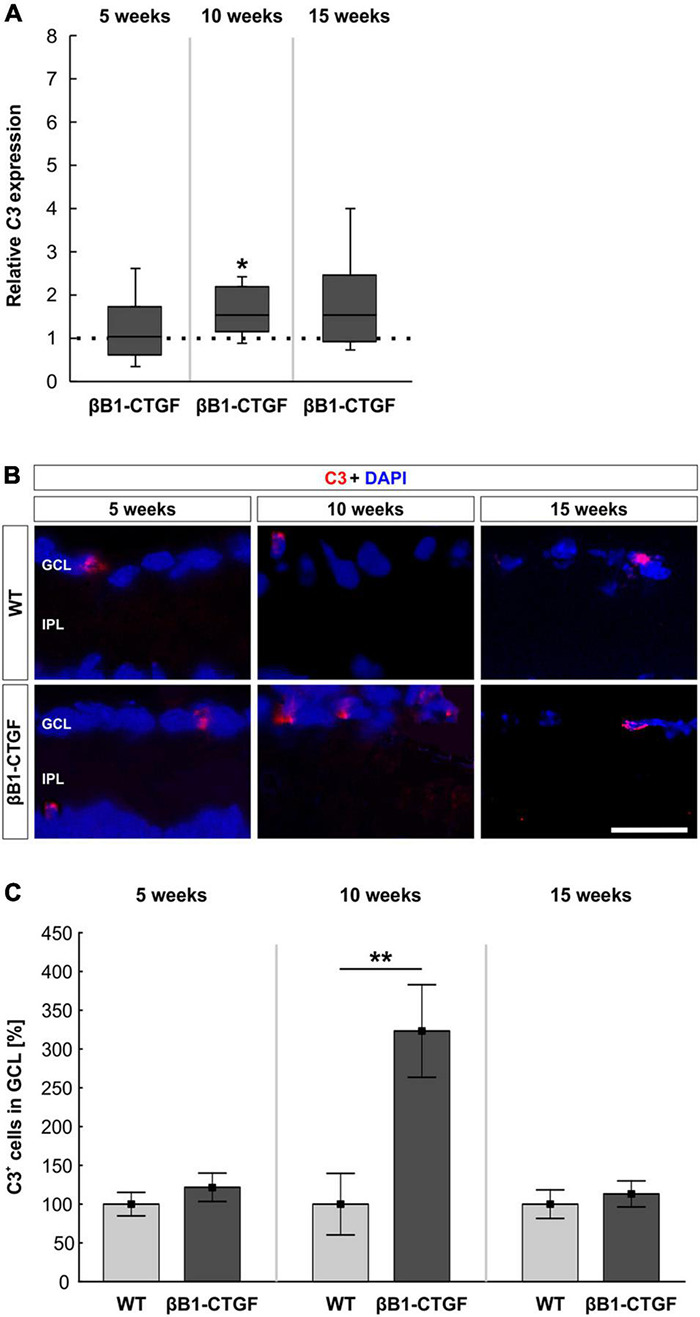
Increased C3 expression in transgenic retinae. **(A)** *C3* mRNA levels were increased in βB1-CTGF retinae at 10 (*p* = 0.048), but not at 5 or 15 weeks. **(B)** Retinal cross-sections were stained with an antibody against C3 (red), while cell nuclei were labeled with DAPI (blue) at all ages. **(C)** The number of C3^+^ cells in the ganglion cell layer was not altered at 5 and 15 weeks. At 10 weeks, significantly more C3^+^ cells could be observed in retinae of βB1-CTGF mice compared to WT (*p* = 0.009). Values for RT-qPCR are median ± quartile ± minimum/maximum (*n* = 5–7/group). The dotted line in panel **(A)** represents the relative expression of the WT group. Values for immunohistology are mean ± SEM (*n* = 7–9/group). GCL, ganglion cell layer; IPL, inner plexiform layer. Scale bar: 20 μm. *p < 0.05, **p < 0.01.

*C3* mRNA levels were evaluated via RT-qPCR at all points in time. In 5-week-old βB1-CTGF mice, no alterations in *C3* mRNA expression levels were noted (1.03-fold expression; *p* = 0.880). At 10 weeks, a significant upregulation of *C3* mRNA could be revealed in transgenic mice (1.55-fold expression; *p* = 0.048). At 15 weeks, the mRNA expression of *C3* was normalized again and remained unchanged (1.55-fold expression; *p* = 0.123; Figure 1A).

Additionally, an anti-C3 antibody was applied to detect depositions in retinal cross-sections (Figure 1B). At 5 weeks, the number of C3^+^ cells remained unchanged in βB1-CTGF mice (121.75 ± 18.27%) compared to WT (100.00 ± 15.22%; *p* = 0.378). Significant more C3^+^ deposits could be detected in the GCL of transgenic animals (323.33 ± 59.69%) in comparison to WT ones (100.00 ± 39.67%; *p* = 0.009) at 10 weeks. The number of C3^+^ cells went back to the control level at 15 weeks (βB1-CTGF: 113.16 ± 16.80%; WT: 100.00 ± 18.37%; *p* = 0.604; Figure 1C).

Further, to gain an insight which cells might produce C3 in the retina, co-stainings with antibodies against anti-NeuN (neurons), anti-GFAP (astrocytes), and anti-Iba1 (macrophages/microglia) were performed in 10-week-old WT and βB1-CTGF mice. A co-localization of C3^+^ cells was mainly found with astrocytes and macrophages/microglia (Supplementary Figures 1A,C,E).

### Enhancement of Terminal Complex Components

**FIGURE 2:**
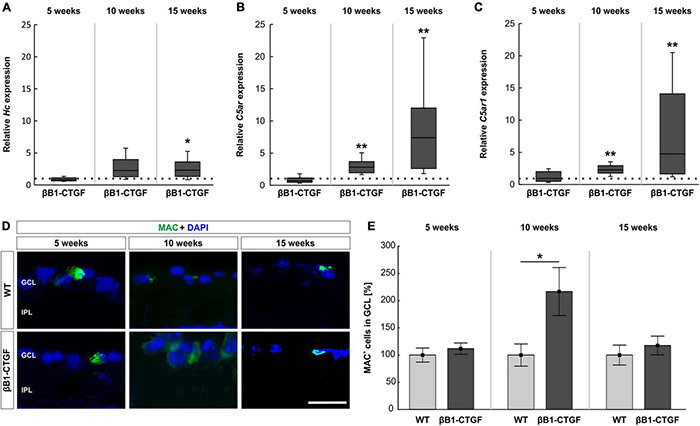
Enhanced terminal pathway components in βB1-CTGF mice. **(A)** The expression of *Hc* (C5) mRNA was not altered in transgenic mice at 5 and 10 weeks. At 15 weeks, a significant upregulation of *Hc* mRNA levels was noted in βB1-CTGF mice (*p* = 0.03). **(B)** RT-qPCR analyses showed no alterations of *C5ar* (C5a anaphylatoxin receptor) mRNA levels at 5 weeks. In contrast, a significant upregulation of *C5ar* mRNA expression levels was detected in 10- (*p* = 0.001) and 15-week-old βB1-CTGF mice (*p* = 0.002). **(C)** The *C5ar1* (complement component C5a receptor 1; CD88) mRNA levels were not altered in 5-week-old transgenic mice, while a significant upregulation was revealed at 10 (*p* = 0.007) and 15 weeks (*p* = 0.001). **(D)** An anti-MAC antibody (green) was applied to label retinal cross-sections at 5, 10, and 15 weeks of age. DAPI counterstained cell nuclei (blue). **(E)** While no changes in the number of MAC^+^ cells were observable at 5 and 15 weeks, a significantly higher number of MAC^+^ cells was noted in 10-week-old transgenic mice (*p* = 0.03). Values for RT-qPCR are median ± quartile ± minimum/maximum (*n* = 5–7/group). The dotted line in panels **(A–C)** represents the relative expression of the WT group. Values for immunohistology are mean ± SEM (*n* = 7–9/group). GCL, ganglion cell layer; IPL, inner plexiform layer. Scale bar: 20 μm. *p < 0.05; **p < 0.001.

The terminal complement pathway consists of several proteins, which in the end assemble MAC. Here, the mRNA expression levels of C5 (*Hc*), as part of MAC, were analyzed through RT-qPCR. With increasing age, the *Hc* mRNA expression levels in transgenic mice continuously enhanced. At 5 weeks, no changes could be noted in *Hc* mRNA expression levels in transgenic mice (0.81-fold expression; *p* = 0.205). In 10-week-old βB1-CTGF mice, a trend toward an upregulation was noted (2.26-fold expression; *p* = 0.059). A significant upregulation of *Hc* mRNA expression levels was observed in 15-week-old transgenic animals compared to WT (2.32-fold expression; *p* = 0.025; Figure 2A).

In addition, we measured the mRNA expression levels of two different receptors for C5a, a potent anaphylatoxin. In particular, the C5a anaphylatoxin receptor (*C5ar*) and the complement component C5a receptor 1 (*C5ar1*, also known as CD88) were analyzed via RT-qPCR. The mRNA expression levels of *C5ar* were not altered in 5-week-old βB1-CTGF mice (0.71-fold expression; *p* = 0.211). The *C5ar* mRNA expression levels were significantly upregulated in transgenic mice at 10 (2.83-fold expression; *p* = 0.007) and 15 weeks of age (7.39-fold expression; *p* = 0.002; Figure 2B). In 5-week-old βB1-CTGF animals, the mRNA expression levels of *C5ar1* (CD88) remained unchanged (0.94-fold expression; *p* = 0.889). A significant upregulation of *C5ar1* mRNA levels was noted in 10- (2.28-fold expression; *p* = 0.007) and 15-week-old βB1-CTGF mice (4.76-fold expression; *p* = 0.001; Figure 2C).

An anti-MAC antibody was used to label retinas at all points in time (Figure 2D). No alterations were noted in βB1-CTGF animals (111.78 ± 10.46%) compared to WT mice (100.00 ± 12.97%; *p* = 0.493) at 5 weeks. In 10-week-old transgenic mice, significantly more MAC^+^ deposits were observed (βB1-CTGF: 216.73 ± 44.11%; WT: 100.00 ± 20.39; *p* = 0.033). The number of MAC^+^ cells were similar in βB1-CTGF mice (117.59 ± 17.20%) and WT animals (100.00 ± 18.27%; *p* = 0.493) at 15 weeks (Figure 2E).

Furthermore, co-stainings with antibodies against anti-NeuN (neurons), anti-GFAP (astrocytes), and anti-Iba1 (macrophages/microglia) in combination with anti-MAC were performed in 10-week-old WT and βB1-CTGF mice. MAC^+^ cells were predominantly co-localized with astrocytes and macrophages/microglia (Supplementary Figures 1B,D,F).

### Activation of Alternative Pathway

**FIGURE 3:**
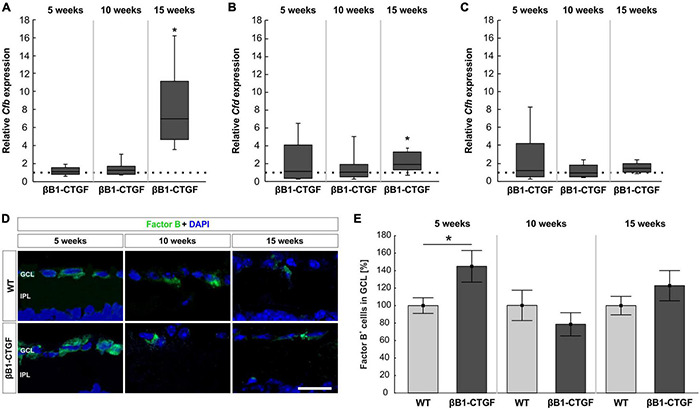
Activation of the alternative pathway. **(A)** At 5 and 10 weeks of age, no alterations in *Cfb* mRNA levels were noted. At 15 weeks, a significant upregulation of *Cfb* mRNA levels was revealed (*p* = 0.01). **(B)** RT-qPCR analyses showed no changes in *Cfd* mRNA levels in 5-and 10-week-old βB1-CTGF mice. In contrast, *Cfd* mRNA levels were significantly upregulated in 15-week-old transgenic mice (*p* = 0.03). **(C)** The mRNA levels of *Cfh* were not altered at 5, 10, and 15 weeks. **(D)** Retinae were stained with an antibody against Factor B (green) and cell nuclei were labeled with DAPI (blue) at all ages. **(E)** The number of Factor B^+^ cells in the ganglion cell layer was significantly higher in βB1-CTGF animals compared to WT (*p* = 0.046). No changes were seen at 10 and 15 weeks. Values for RT-qPCR are median ± quartile ± minimum/maximum (*n* = 5–7/group). The dotted line in panels **(A–C)** represents the relative expression of the WT group. Values for immunohistology are mean ± SEM (*n* = 7–9/group). GCL, ganglion cell layer; IPL, inner plexiform layer. Scale bar: 20 μm. *p < 0.05.

The activators of the alternative complement pathway, *Cfb* and *Cfd* as well as the regulator *Cfh* were examined via RT-qPCR analysis. At 5 and 10 weeks, no alterations were noted in *Cfb* mRNA expression levels in βB1-CTGF retinae compared to WT (5 weeks: 1.11-fold expression; *p* = 0.542; 10 weeks: 1.27-fold expression; *p* = 0.173). In 15-weeks old transgenic mice, *Cfb* mRNA expression levels were significantly upregulated (7.01-fold expression; *p* = 0.013; Figure 3A).

The mRNA expression levels of *Cfd* remained unchanged in 5- (1.13-fold expression; *p* = 0.813) and 10-week-old βB1-CTGF mice (1.04-fold expression; *p* = 0.864). However, a significant upregulation of *Cfd* mRNA levels was noted in 15-week-old transgenic animals (1.96-fold expression; *p* = 0.034; Figure 3B).

The *Cfh* mRNA expression levels were not altered in transgenic mice at 5 (1.16-fold expression; *p* = 0.781), 10 (0.93-fold expression; *p* = 0.712), and 15 weeks (1.40-fold expression; *p* = 0.105; Figure 3C).

In addition, retinal cross-sections were labeled with an antibody against Factor B at all points in time (Figure 3D). At 5 weeks, significantly more Factor B^+^ cells were counted in the GCL of transgenic mice (144.91 ± 18.11%) compared to WT (100.00 ± 8.96%; *p* = 0.046). At 10 weeks, the number of Factor B^+^ cells in βB1-CTGF retinae (78.47 ± 13.24%) was comparable with WT ones (100.00 ± 17.31%; *p* = 0.348). Furthermore, no alterations in Factor B cell counts were observed in 15-week-old transgenic animals (122.79 ± 17.28%) in contrast to WT (100.00 ± 10.55%; *p* = 0.282; Figure 3E).

### Involvement of Classical Route

**FIGURE 4:**
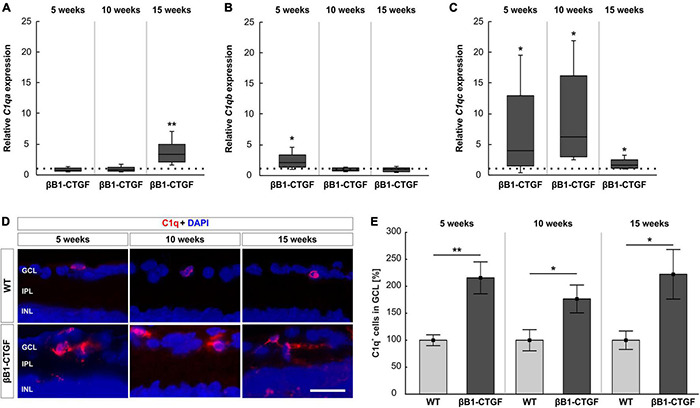
C1q upregulation in transgenic animals. **(A)** RT-qPCR analyses revealed no alterations in *C1qa* mRNA levels in βB1-CTGF mice at 5 and 10 weeks. At 15 weeks, a significant upregulation of *C1qa* mRNA levels was seen (*p* = 0.005). **(B)** At 5 weeks, *C1qb* mRNA levels were significantly upregulated (*p* = 0.04), while no changes were noted at 10 and 15 weeks. **(C)** The mRNA levels of *C1qc* were significantly upregulated in transgenic mice at 5 (*p* = 0.04), 10 (*p* = 0.01), and 15 weeks of age (*p* = 0.03). **(D)** Retinal cross-sections were labeled with an antibody against C1q (red), while DAPI counterstained cell nuclei (blue) in 5-, 10-, and 15-week-old mice. **(E)** The number of C1q^+^ cells in the ganglion cell layer was significantly higher in βB1-CTGF and WT animals at 5 (*p* = 0.003), 10 (*p* = 0.04), and 15 weeks (*p* = 0.03). Values for RT-qPCR are median ± quartile ± minimum/maximum (*n* = 5–7/group). The dotted line in panels **(A–C)** represents the relative expression of the WT group. Values for immunohistology are mean ± SEM (*n* = 7–9/group). GCL, ganglion cell layer; IPL, inner plexiform layer; INL, inner nuclear layer. Scale bar: 20 μm. *p < 0.05, **p < 0.01.

The mRNA expression levels of components of C1q, namely *C1qa*, *C1qb*, and *C1qc*, were assessed via RT-qPCR analysis. In 5- and 10-week-old βB1-CTGF mice, no alterations were noted in *C1qa* mRNA expression levels (5 weeks: 0.77-fold expression; *p* = 0.223; 10 weeks: 0.82-fold expression; *p* = 0.400). However, a significant upregulation of *C1qa* mRNA was observable in 15-week-old transgenic animals (3.12-fold expression; *p* = 0.005; Figure 4A). Regarding the mRNA expression levels of *C1qb*, a significant enhancement was noted in 5-week-old βB1-CTGF mice (2.04-fold expression; *p* = 0.041), but not at 10 (0.84-fold expression; *p* = 0.247) and 15 weeks (0.92-fold expression; *p* = 0.550; Figure 4B). The mRNA expression levels of *C1qc* were significantly upregulated in 5-week-old transgenic animals (3.99-fold expression; *p* = 0.044). Also, at 10 and 15 weeks, a significant enhancement of *C1qc* mRNA levels was observed in βB1-CTGF mice (10 weeks: 6.17-fold expression; *p* = 0.014; 15 weeks: 1.63-fold expression; *p* = 0.034; Figure 4C).

Besides RT-qPCRs, an anti-C1q antibody was utilized to label retinal cross-sections at 5, 10, and 15 weeks of age (Figure 4D). At 5 weeks, the number of C1q^+^ cells was significantly higher in βB1-CTGF retinae (215.72 ± 29.57%) compared to WT animals (100.00 ± 10.06%; *p* = 0.003). Further, significantly more C1q^+^ cells could be detected at 10 (βB1-CTGF: 176.61 ± 25.82%; WT: 100.00 ± 19.63; *p* = 0.036) and 15 weeks (βB1-CTGF: 222.28 ± 45.95%; WT: 100.00 ± 17.06; *p* = 0.028; Figure 4E).

### Mild Activation of Lectin Pathway

**FIGURE 5:**
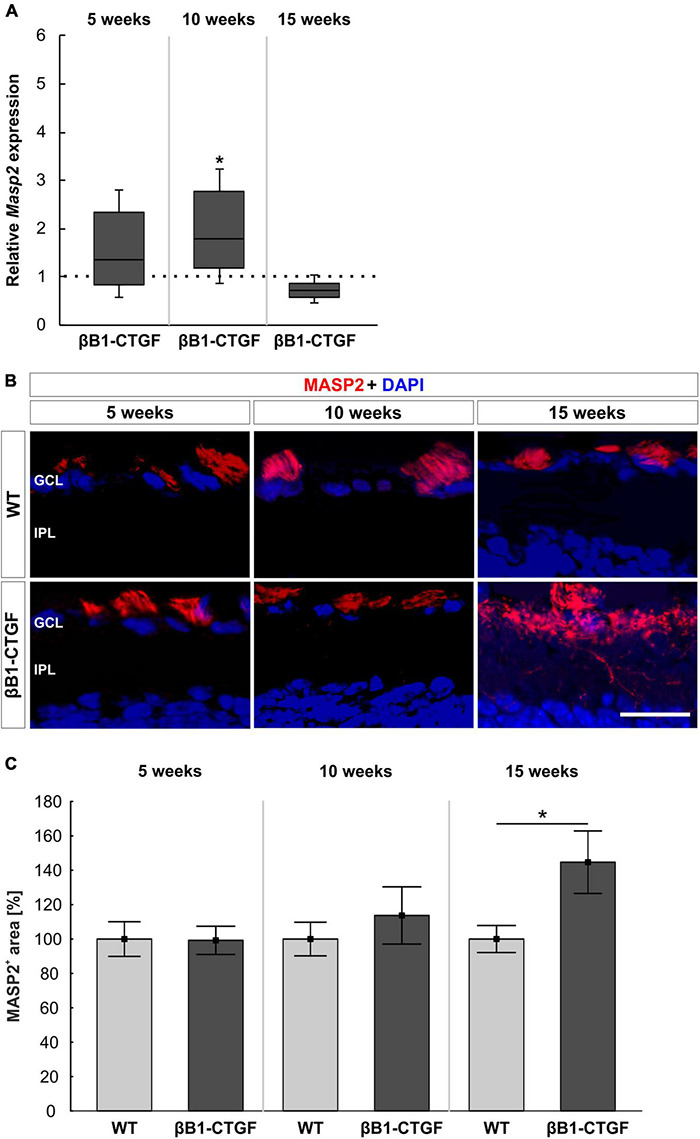
Lectin pathway is initiated. **(A)** The mRNA expression levels of *Masp2* were upregulated at 10 (*p* = 0.047), but not at 5 and 15 weeks, in βB1-CTGF mice. **(B)** An anti-MASP2 antibody (red) was used to label retinal cross-sections at 5, 10, and 15 weeks of age. Cell nuclei are counterstained with DAPI (blue). **(C)** The MASP2^+^ area was not altered in transgenic mice compared to WT at 5 and 10 weeks. At 15 weeks, a significant increase of MASP2^+^ area was revealed in βB1-CTGF animals (*p* = 0.04). Values for RT-qPCR are median ± quartile ± minimum/maximum (*n* = 5–7/group). The dotted line in panel **(A)** represents the relative expression of the WT group. Values for immunohistology are mean ± SEM (*n* = 7–9/group). GCL, ganglion cell layer; IPL, inner plexiform layer. Scale bar: 20 μm. *p < 0.05.

*Masp2* mRNA expression levels were analyzed through RT-qPCR. At 5 weeks, the *Masp2* mRNA expression was not altered in βB1-CTGF mice compared to WT (1.35-fold expression; *p* = 0.297). In 10-week-old transgenic animals, a significant upregulation of *Masp2* mRNA was noted (1.79-fold expression; *p* = 0.047). At 15 weeks, the mRNA expression of *Masp2* went back to WT level (0.72-fold expression; *p* = 0.077; Figure 5A).

Additionally, retinal cross-sections were labeled with an anti-MASP2 antibody at all points in time (Figure 5B). It was noted that the MASP2^+^ area remained unchanged in 5- and 10-week-old βB1-CTGF animals in contrast to WT ones (5 weeks: βB1-CTGF: 99.25 ± 8.21%; WT: 100.00 ± 10.07; *p* = 0.955; 10 weeks: βB1-CTGF: 113.71 ± 16.63%; WT: 100.00 ± 9.78%; *p* = 0.489). At 15 weeks, a significant increase of MASP2^+^ area was observed in transgenic mice (144.70 ± 18.19% compared to WT (100.00 ± 7.86%; *p* = 0.044; Figure 5C).

### Enhanced Cytokine Activation

RT-qPCRs were performed to identify the mRNA levels of INF-γ (*Infg*), the two murine homologs of IL8 CXCL-1 (*Cxcl1*) and CXCL-2/MIP-2 (*Cxcl2*) (Bozic et al., 1994; Hol et al., 2010) as well as CXCL-10 (*Cxcl10*) at all ages.

**FIGURE 6:**
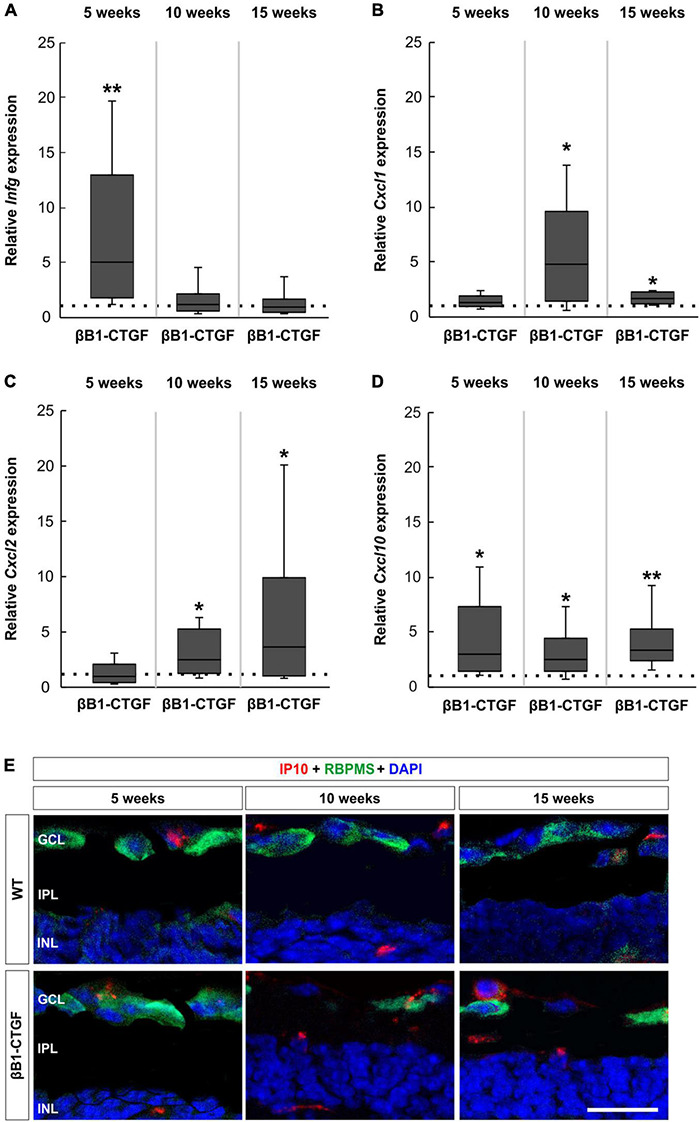
Upregulated cytokine expression in βB1-CTGF retinae. **(A)** RT-qPCR analyses showed an upregulation of *Infg* mRNA levels in βB1-CTGF retinae at 5 (*p* = 0.009), but not at 10 and 15 weeks of age. **(B)** The mRNA expression levels of *Cxcl1* were not altered in 5-week-old transgenic mice. At 10 and 15 weeks, significantly higher mRNA levels of *Cxcl1* were noted in βB1-CTGF mice (10 weeks: *p* = 0.01; 15 weeks: *p* = 0.04). **(C)** RT-qPCR analyses revealed no changes in the mRNA expression levels of *Cxcl2* in 5-week-old transgenic animals. A significant upregulation of *Cxcl2* mRNA levels was noted in βB1-CTGF mice at 10 (*p* = 0.03) and 15 weeks (*p* = 0.04). **(D)** *Cxcl10* mRNA levels were significantly upregulated in βB1-CTGF retinae at 5 (*p* = 0.03), 10 (*p* = 0.02), and 15 weeks (*p* = 0.002). **(E)** Representative pictures of retinal cross-sections labeled with an antibody against IP-10 (CXCL-10; red) and RBPMS (RGCs; green), while DAPI counterstained cell nuclei (blue). IP-10^+^ cells were predominantly observed in the ganglion cell layer and inner nuclear layer of βB1-CTGF mice. Values are median ± quartile ± minimum/maximum (*n* = 5–6/group). The dotted line in panels **(A–D)** represents the relative expression of the WT group. GCL, ganglion cell layer; IPL, inner plexiform layer; INL, inner nuclear layer. Scale bar: 20 μm. *p < 0.05, **p < 0.01.

*Infg* mRNA expression levels were upregulated in 5-week-old βB1-CTGF mice compared to WT (5.01-fold expression; *p* = 0.009). The *Infg* mRNA expression went back to WT levels in 10- (1.11-fold expression; *p* = 0.780) as well as in 15-week-old βB1-CTGF animals (0.90-fold expression; *p* = 0.807; Figure 6A).

Regarding *Cxcl1* mRNA expression levels, no changes could be noted in 5-week-old transgenic mice (1.22-fold expression; *p* = 0.341). However, a significant increase of *Cxcl1* mRNA expression levels was observed at 10 weeks (3.75-fold expression; *p* = 0.013). Also, *Cxcl1* mRNA levels were upregulated in 15-week-old transgenic animals compared to WT (1.61-fold expression; *p* = 0.027; Figure 6B).

The mRNA expression levels of *Cxcl2* were not altered in 5-week-old transgenic mice (0.98-fold expression; *p* = 0.940). At 10 weeks, a significant upregulation of *Cxcl2* mRNA levels was noted in βB1-CTGF mice (2.49-fold expression; *p* = 0.031). In addition, a significant upregulation of *Cxcl2* mRNA was revealed in 15-week-old transgenic animals (3.64-fold expression; *p* = 0.038; Figure 6C).

The analysis of *Cxcl10* mRNA expression levels revealed a significant upregulation in 5-week-old βB1-CTGF mice in contrast to WT ones (2.96-fold expression; *p* = 0.029). The *Cxcl10* mRNA expression levels remained upregulated at 10 (2.50-fold expression; *p* = 0.017) as well as at 15 weeks (3.36-fold expression; *p* = 0.002; Figure 6D).

Retinal cross-sections were labeled with an antibody against IP-10 (CXCL-10) and RBPMS. Representative pictures show that CXCL-10^+^ cells were predominantly localized in the GCL and inner nuclear layer of βB1-CTGF mice (Figure 6E).

## Discussion

Glaucoma is a complex neurodegenerative disease, which can lead to blindness when untreated. It often remains asymptomatic for a long time, since progressive visual field loss is peripheral and typically asymmetric, which allows for compensation from the overlapping visual field of the other eye (Gupta and Chen, 2016). Unfortunately, the only currently modifiable factor is the IOP. Therefore, there is an urgent need for new and additional therapeutic approaches. Hence, other mechanisms besides the main risk factor IOP elevation need to be investigated.

For instance, previous studies concluded that an activation of the complement system seems to contribute to cell death in glaucoma. In the study presented here, we aimed to determine complement activation as well as cytokine expression in different ages of the transgenic βB1-CTGF mouse model. This model enables to study underlying pathomechanisms occurring in high-pressure glaucoma. In contrast to other OHT models, no surgical interventions are needed. While the IOP was not altered in 5- and 10-week-old βB1-CTGF mice, elevated IOP was accompanied by an apoptotic loss of RCGs in 15-week-old animals (Reinehr et al., 2019b). Furthermore, more apoptotic RGCs and TUNEL^+^ apoptotic cells were already noted in 5- and 10-week-old transgenic mice (Weiss et al., 2021). The relation of apoptotic cells and complement activation should be investigated more precisely in future studies.

In the aqueous humor of patients suffering from POAG, the complement factor C3 was elevated (Liu et al., 2020). Recently, Hubens et al. (2021) demonstrated an upregulation of the C3a/C3 ratio in aqueous humor from progressive, but not from stable POAG patients. In our study, we could demonstrate that the terminal complement pathway components C3 and MAC were increased in 10-week-old βB1-CTGF mice before an RGC death was noted. Especially, the formation of MAC could lead to the apoptosis of cells, which was shown, for example, in rat mesangial cells (Nauta et al., 2002). Regarding the initiation of the complement system, similar results were obtained in an IOP-independent experimental autoimmune glaucoma (EAG) model. Here, immunization with ocular antigens led to an activation of the complement system before RGC and optic nerve degeneration (Reinehr et al., 2016, 2018b). Also, in DBA/2J.*Wld*^5^ mice, which are protected from axon dysfunction, early C3 upregulation could be observed (Harder et al., 2017). Further, the C5a anaphylatoxin receptor (*C5ar*) as well as the complement component C5a receptor 1 (*C5ar1;* CD88) were found upregulated transgenic mice at 10 and 15 weeks of age. C5a itself is a powerful mediator of inflammation and is important in the recruitment and chemotaxis of glia to the site of damage, through its anaphylatoxin G-protein coupled receptor (Yao et al., 1990; Miller and Stella, 2009; Woodruff et al., 2010).

In accordance with studies in other glaucoma models, our results reveal that complement activation is an early event in glaucoma pathology.

To elaborate, which cells might be the source of C3 and MAC in our model, we performed co-stainings with markers for neuronal cells, astrocytes, and macrophages/microglia in 10-week-old WT and βB1-CTGF mice. These stainings show that, most likely, astrocytes and/or macrophages/microglia are the main source of local complement in our model. After light-induced damage and in the aging retina, microglia were identified to express the complement component C3 (Rutar et al., 2011, 2014). In an Alzheimer’s disease mouse model, the authors showed that under physiological conditions, C3 is expressed primarily by astrocytes (Lian et al., 2016). Another study revealed a direct involvement of Mueller cells in the transcript expression of the retinal complement components, and it is likely that in the retina neurons and glia cells orchestrate complement-mediated maturation (Tenner et al., 2018; Pauly et al., 2019). Nonetheless, the origin and sources of the complement proteins in the βB1-CTGF model should be explored in more detail in further experiments.

As mentioned, the complement system can be initialized through three different pathways. In the βB1-CTGF mouse model, we observed an activation of all three pathways throughout the study. Especially C1q, as a member of the classical route, was upregulated at all investigated ages, namely in 5-, 10-, and 15-week-old βB1-CTGF mice. Activation of the classical pathway was also previously described in other glaucoma models. For example, Stasi et al. detected that a C1q upregulation in the retina of DBA/2J mice preceded RGC death (Stasi et al., 2006). After injection of microbeads in mice, which leads to OHT, elevated levels of C1q mRNA were detected (Krishnan et al., 2019). Also, in rats with OHT, elevated C1q levels were noted (Kuehn et al., 2006). Additionally, more C1q was found in laser-induced glaucomatous monkey eyes (Stasi et al., 2006). Furthermore, Howell et al. (2011) showed that mice with a C1q mutation were protected from glaucomatous damage. But not only in animal models, also in human glaucoma donor eyes, C1q depositions were observed (Stasi et al., 2006). Hence, the classical pathway plays an important role in complement system activation during glaucomatous damage.

Interestingly, we also observed an upregulation of the lectin pathway component MASP2 via RT-qPCR and immunohistology. Although the lectin pathway recognizes carbohydrate structures mostly on bacteria, viruses, fungi, and parasites, it is also able to bind to similar structures on apoptotic or necrotic cells (Neth et al., 2000; Turner, 2003; Stuart et al., 2005). This was previously also noted in the EAG model, where IOP is not increased. Here, an activation of the lectin pathway was observed (Reinehr et al., 2016, 2018b). In addition, increased levels of several complement components, including MASP2 were found in glaucoma patients (Tezel et al., 2010). Furthermore, in sera of POAG patients, higher levels of the mannose binding lectin 2 were detected (Dursun et al., 2012).

The alternative pathway of the complement system is spontaneously activated and often associated with processes occurring in age-related macular degeneration (Cipriani et al., 2020). Regarding glaucoma, little is known about the contribution of this pathway. In our βB1-CTGF mice, more Factor B^+^ cells were counted in the GCL at 5 weeks, while enhanced mRNA levels of *Cfb* and *Cfd* were observed at 15 weeks. Recently, Fernandez-Vega Cueto et al. (2020) investigated several immune components in sera of DBA2/J mice and their ability as biomarker. They noted that a five protein-panel, amongst others this included complement factor H, as another component of the alternative pathway, predicted the transition to glaucoma in about 78% of these animals (Fernandez-Vega Cueto et al., 2020). Also, in the IOP-independent EAG model an early upregulation of *Cfb* mRNA levels was observed (Reinehr et al., 2018b).

It is notable to mention the discrepancy of some results regarding immunohistological and RT-qPCR findings. For example, the results of the alternative and lectin complement pathway seem to differ between the two methods. Immunohistological stainings, particularly in the retina, have the advantage to show the distribution of different cell types within the retinal layers. Further, with this method we were able to count complement positive cells only in the GCL since this is the most affected one during glaucoma. In contrary, for RT-qPCR analyses, RNA and cDNA syntheses were performed using the whole retina. This could explain the discrepancy in the results. In addition, the inconsistency could be explained with different posttranscriptional and translational regulations. Nonetheless, using both methods enabled us to determine the complement response in βB1-CTGF mice quite well.

Overall, these results reveal that the complement system is active in early stages in βB1-CTGF mice and especially the classical pathway plays a role in its initiation. Therefore, therapeutic approaches should be focused on components like C5 to inhibit the terminal pathway, as shown in previous studies (Howell et al., 2013; Reinehr et al., 2019a; Gassel et al., 2021).

Complement activation not only harms cells directly, but its activation is also engaged in the regulation of an inflammatory reaction (Markiewski and Lambris, 2007). C5a, for example, can act directly on neutrophils and monocytes to increase their ability to adhere to vessel walls, their migration toward sites of antigen deposition as well as to ingest particles (Murphy et al., 2008). In osteoblasts, C5a induced migration and expression of IL-8 (Ignatius et al., 2011). Furthermore, C5aR1, in interaction with toll-like-receptor 2, resulted in an upregulation of CXCL-10 (Modinger et al., 2018). We could determine that the mRNA levels of the examined C5a receptors, *C5ar* and *C5ar1*, were significantly upregulated in 10- and 15-week-old transgenic animals. An upregulation of *C5ar* was also noted in mouse retinae 2 days after light damage and a C5aR knockout was able to diminish microglia cells (Song et al., 2017). Additionally, Zhang et al. showed that C3aR/C5aR double knockout mice developed less severe uveitis, which supports the role of these receptors in retinal inflammation (Zhang et al., 2016). Further, we found upregulated *Cxlc10* mRNA levels in βB1-CTGF retinae at 5, 10, and 15 weeks of age, while an elevation of *Cxcl1* and *Cxcl2* (IL-8) mRNA levels was noted at 10 and 15 weeks. At 5 weeks, an upregulation of *Infg* was also demonstrated in transgenic mice. iPS-retinal pigment epithelium (RPE) cells can express complement factors, such as C3, C5, and MAC, especially after INF-γ exposure (Sugita et al., 2018). RPE cells with activated T-cells express INF-γ associated chemokines, such as CXCL-10 (Sugita et al., 2006). Under the influence of IFN-γ, CXCL-10 is secreted by several cell types including endothelial cells, fibroblasts, keratinocytes, thyrocytes, or preadipocytes (Antonelli et al., 2014). It seems likely that the upregulation of these cytokines correlates with the activation of the complement system and contribute to cell death in transgenic βB1-CTGF mice.

## Conclusion

**FIGURE 7:**
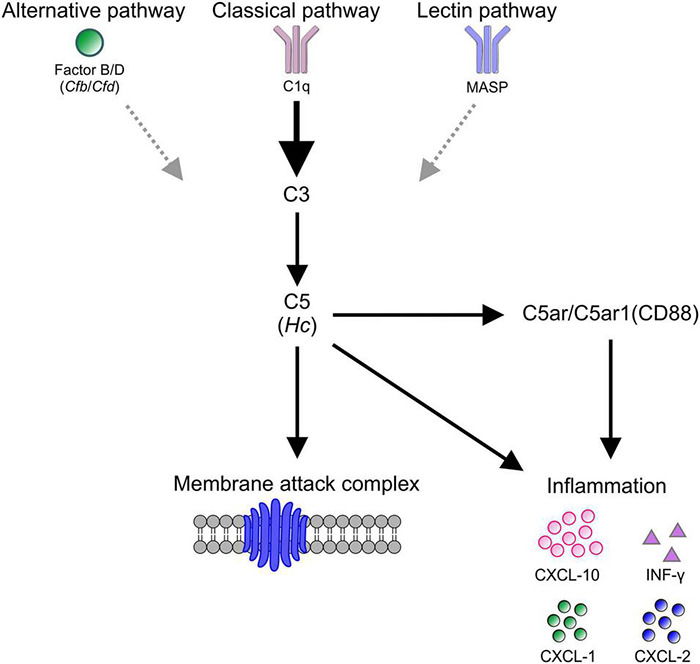
Schematic overview of study findings. In βB1-CTGF mice, which develop glaucomatous damage, an activation of the complement, predominantly via the classical pathway (C1q), could be detected. The activation of C5 (*Hc*) eventually lead to the assembling of the membrane attack complex (MAC), which harms retinal ganglion cells directly. Further, C5 as well as C5a receptors, namely the C5a anaphylatoxin receptor (*C5ar*) and the complement component C5a receptor 1 (*C5ar1*, CD88), engage a pro-inflammatory response of INF-γ, CXCL-10, CXCL-1, and CXCL-2.

Cell death in βB1-CTGF mice seems to be associated with an activation of the complement system, especially through the classical pathway and corresponding cytokine response (Figure 7). These results further support the hypothesis that an altered immune response plays a crucial role in glaucomatous neurodegeneration.

## Data Availability Statement

The raw data supporting the conclusions of this article will be made available by the authors, without undue reservation.

## Ethics Statement

The animal study was reviewed and approved by the Landesamt für Natur, Umwelt und Verbraucherschutz Nordrhein-Westfalen.

## Author Contributions

SR performed the experiments, analyzed the data, and wrote the manuscript. JD, AM-B, and DK performed the experiments and analyzed the data. RF and HD revised the manuscript. SJ conceived the study and revised the manuscript. All authors read and approved the final manuscript.

## Conflict of Interest

The authors declare that the research was conducted in the absence of any commercial or financial relationships that could be construed as a potential conflict of interest.

## Publisher’s Note

All claims expressed in this article are solely those of the authors and do not necessarily represent those of their affiliated organizations, or those of the publisher, the editors and the reviewers. Any product that may be evaluated in this article, or claim that may be made by its manufacturer, is not guaranteed or endorsed by the publisher.
