# Supplementary material for: Cytokine and Complement Response in the Glaucomatous βB1-CTGF Mouse Model
Source: Front Cell Neurosci. 2021 Nov 18;15:718087. doi: 10.3389/fncel.2021.718087 (PMC8637215; doi:10.3389/fncel.2021.718087)

**Supplementary Figure 1: Co-localization of complement factors with astrocytes and macrophages/microglia. A)** Retinal cross-sections of 10-week-old wildtype (WT) and transgenic βB1-CTGF mice were labeled with anti-C3 (green) and anti-NeuN (red; neurons) antibodies, while DAPI (blue) counterstained cell nuclei. C3^+^ cells seemed not be co-localized with NeuN^+^ cells. **B)** At 10 weeks, retinae were stained with anti-MAC (green) and anti-NeuN (red). DAPI labeled cell nuclei (blue). As for C3, MAC^+^ cells were not co-localized with NeuN^+^ neuronal cells in WT and βB1-CTGF mice. **C)** WT and βB1-CTGF retinae were stained with antibodies against C3 (green) and GFAP (red; astrocytes). Cell nuclei were counterstained with DAPI (blue). In 10-week-old animals, C3^+^ cells were often co-localized with astrocytes (arrows), especially in transgenic mice. **D)** Anti-MAC (green) and anti-GFAP (red) antibodies were used to label retinal cross-sections of 10-week-old WT and βB1-CTGF, while DAPI was used to visualize cell nuclei (blue). MAC^+^ cells were also found often co-localized with GFAP^+^ astrocytes (arrows). **E)** At 10 weeks, retinae were stained with anti-C3 (green) and anti-Iba1 antibodies (red; macrophages/microglia). DAPI (blue) counterstained cell nuclei. Particularly in transgenic mice, some C3^+^ cells were co-localized with Iba1^+^ macrophages/microglia (arrow). **F)** Anti-C3 (green) and anti-Iba1 (red) antibodies were applied onto retinal cross-sections of WT and βB1-CTGF mice at 10 weeks of age, while DAPI (blue) labeled cell nuclei. As seen for C3, some MAC^+^ cells were in co-localization with Iba1^+^ macrophages/microglia (arrow). GCL=ganglion cell layer; IPL=inner plexiform layer; INL=inner nuclear layer. Scale bars: 20 µm.


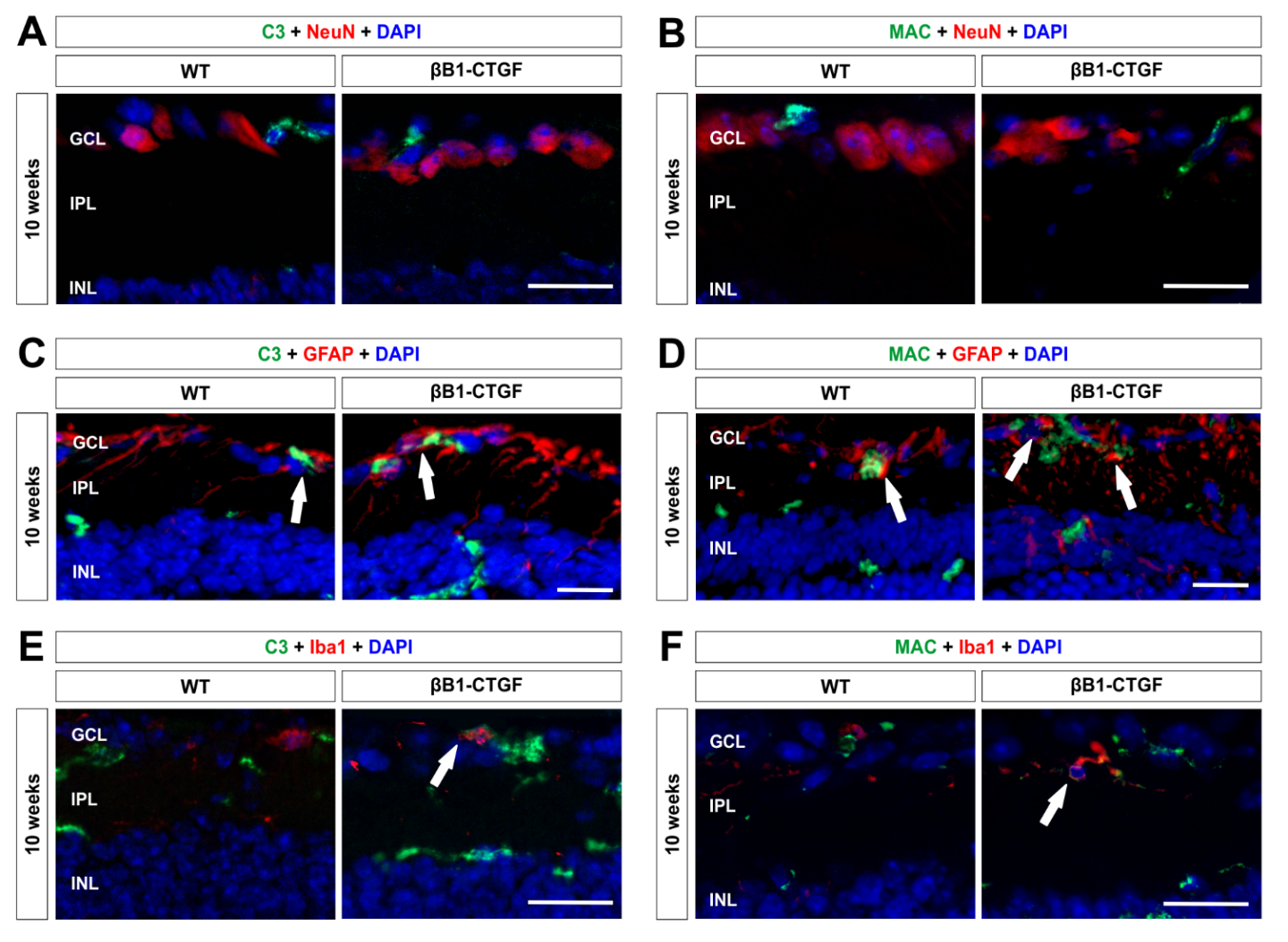

Supplement: Supplementary file 1 [file Data_Sheet_1.docx]
